# Systemic Autoimmune Diseases 2014

**DOI:** 10.1155/2015/183591

**Published:** 2015-05-24

**Authors:** Guixiu Shi, Jianying Zhang, Zhixin (Jason) Zhang, Xuan Zhang

**Affiliations:** ^1^Department of Rheumatology and Clinical Immunology, The First Hospital of Xiamen University, Xiamen 361003, China; ^2^Laboratory of Autoimmunity, School of Biological Sciences, University of Texas at El Paso, El Paso, TX 79968, USA; ^3^Department of Pathology and Microbiology, College of Medicine, University of Nebraska Medical Center, Omaha, NE 68198-7660, USA; ^4^Department of Rheumatology, Peking Union Medical College Hospital, Beijing 100032, China

Systemic autoimmune diseases are a group of common diseases, including rheumatoid arthritis, systemic lupus erythematosus, spondyloarthropathy, Sjogren's syndrome, polymyositis, and dermatomyositis, etc. They are one of the leading causes of death and disability. With the use of glucocorticoid, immune suppression drugs and new developed biologics, the outcome of this group of diseases has greatly improved, but there is still no cure for them. Knowledge of the pathogenesis, diagnosis, and treatment of those diseases will lead to better understanding of the diseases and better care of patients.

Based on this background, we assembled this special issue for a better understanding of the molecular pathology underlying systemic autoimmune diseases, the development of strategies to treat these conditions, and the evaluation of outcomes.

In this special issue, several review articles discussed many important aspects about autoimmune disease. A. Mastrangelo et al. discussed the role of posttranslational protein modifications in rheumatoid arthritis. C. Gluhovschi et al. made a review about pregnancy associated with systemic lupus erythematosus. A. Kronbichler et al. reviewed the influence and role of microbial factors in autoimmune kidney diseases. T. Shizuma summarized clinical characteristics of concomitant systemic lupus erythematosus and primary biliary cirrhosis. Z. Wu and H. Nakanishi discussed the link between inflammatory bone disorders and Alzheimer's disease. K. R. Sigdel et al. made a review about the functions of long noncoding RNA in immune cells. L. Duan et al. made a review about treatment of bullous systemic lupus erythematosus. R. Hage-Sleiman et al. reviewed recent studies about the novel PKCtheta. L. Zhang et al. made a meta-analysis about interleukin-23R rs7517847 T/G polymorphism and the risk of Crohn's disease in Caucasians.

Beside reviews, many original research studies about the pathogenesis or clinical characteristics of autoimmune disease were also included in this special issue. T. Elisa et al. studied the role of endothelin receptors in the pathogenesis of systemic sclerosis. A. Barbieri et al. analyzed the characterization of CD30/CD30L+ cells in peripheral blood and synovial fluid of patients with rheumatoid arthritis. G. F. Dong et al. researched the effect of leflunomide on the lipid rafts expression in SLE patients. P. Žigon et al. found that anti-phosphatidylserine/prothrombin antibodies were associated with adverse pregnancy outcomes. G. Sudzius et al. studied the distribution of peripheral lymphocyte populations in primary Sjögren's syndrome patients. J. Xu et al. showed that autoantibodies affect brain density reduction in nonneuropsychiatric systemic lupus erythematosus patients. B. Shen et al. found that body image disturbances have impact on the sexual problems in systemic lupus erythematous patients. G. Guo et al. and C. Zhao et al. studied mesenchymal stem cells in SLE and RA patients. Y. Liu et al. found a new serological marker in SLE patients. A. E. Ngono et al. found that frequency of circulating myelin oligodendrocyte glycoprotein B lymphocytes was decreased in relapsing-remitting multiple sclerosis patients. J. Amaya-Amaya et al. showed that GDF15 (MIC1) H6D polymorphism does not influence cardiovascular disease in a Latin American population with rheumatoid arthritis. A. D. Rocha-Muñoz et al. demonstrated that anti-CCP2 antibodies are markers associated with the severity of RA- ILD. M. Lu et al. found that HMGB1 promoted systemic lupus erythematosus by enhancing macrophage inflammatory response. A. D. Rocha-Muñoz et al. studied the influence of anti-TNF and disease modifying antirheumatic drugs (DMARDs) therapy on pulmonary forced vital capacity associated with ankylosing spondylitis. B. Kisiel et al. showed that methotrexate, cyclosporine A, and biologics protect against atherosclerosis in rheumatoid arthritis. L. Wang et al. analyzed clinical characteristics of cerebral venous sinus thrombosis in SLE patients.

This special issue covers many important aspects in autoimmune diseases, which will surely provide us with a better understanding about the pathogenesis, diagnosis, and treatment of autoimmune diseases.

*Guixiu Shi* *Guixiu Shi* *Jianying Zhang* *Jianying Zhang* *Zhixin (Jason) Zhang* *Zhixin (Jason) Zhang* *Xuan Zhang* *Xuan Zhang*

